# Management of acute exacerbations in multiple sclerosis

**DOI:** 10.4103/0972-2327.58283

**Published:** 2009

**Authors:** Daniel Ontaneda, Alex D. Rae-Grant

**Affiliations:** Neurological Institute, Cleveland Clinic, Cleveland, Ohio, 44195 USA

**Keywords:** Exacerbation, management, multiple sclerosis, relapse, steroids

## Abstract

A key component of multiple sclerosis is the occurrence of episodes of clinical worsening with either new symptoms or an increase in older symptoms over a few days or weeks. These are known as exacerbations of multiple sclerosis. In this review, we summarize the pathophysiology and treatment of exacerbations and describe how they are related to the overall management of this disease.

## Introduction

In the past 20 years, the focus in multiple sclerosis (MS) management has shifted away from the care of patients during subacute worsening of their disease to the prevention of new disease activity. This is not surprising given the multiple agents that are now available for disease management. Still, the treatment of acute exacerbations of MS remains an important component of clinical care for this population and should be embedded in an understanding of the significance and pathophysiology of such events.

Approximately 80% of all multiple sclerosis (MS) patients present, initially, with a relapsing form of the disease.[1] Exacerbations continue to occur throughout the relapsing remitting stage of MS. It is during this stage of the disease that incomplete resolutions of relapses accrue over time and result in disability.[2] A high frequency of exacerbations in the first year after diagnosis is also a predictor of worse outcome.[3] Aggressive treatment for the prevention of exacerbations has therefore been the main goal of disease modifying therapy in relapsing remitting MS (RRMS). Treatment of exacerbations, once they occur, has been directed at shortening duration of the attack and promoting a complete recovery. This has typically been accomplished with anti-inflammatory agents such as steroids and ACTH. Great advances have been made in our ability to reduce the incidence of relapses with introduction of multiple medications and active research with new agents. However, the treatment of exacerbations has suffered no major changes in the last 20 years and has not been the major focus of research in the MS field. The full scope of the effect of exacerbations on disease progression, disease related cost, and psychological effects for MS patients has yet to be fully described. In this review, we focus on the management of acute exacerbations. Disease modifying agents are discussed elsewhere in this supplement.

## Definition of Exacerbations

Exacerbations have been typically defined as episodes of focal neurological disturbance lasting more than 24 h, without an alternate explanation, and with a preceding period of clinical stability lasting at least 30 days.[4] Fluctuations in symptoms or worsening of symptoms with fever, heat, or infection are not considered true exacerbations unless they meet the above criteria. They are often referred to as pseudo-exacerbations.[5] It is to be noted, however, that infections tend to increase the risk of an exacerbation and lengthen the total duration of these as well.[6] Other terms that have been used for exacerbations include relapses, attacks, and bouts. These terms are synonymous.

## Biological basis of exacerbations

Relapses in multiple sclerosis have been attributed to the occurrence of new white matter lesions. This was first demonstrated with magnetic resonance imaging (MRI) studies showing gadolinium enhanced white matter lesions in patients with relapses.[7] MS lesions are felt to result from a loss of integrity in the blood brain barrier with subsequent migration of immune reactive cells that target myelin and oligodendrocytes.[8]

The exact factors governing the initial disruption of the blood brain barrier are not entirely understood; however, altered chemokine release and expression is assumed to play a role.[9] Trafficking of CD4+ CD8+ T cells into the CNS is the next step of activation. The inflammatory response in MS lesions is primarily a T-Cell mediated response. In the animal model of MS- Experimental Autoimmune Encephalomyelitis (EAE), there appears to be an early predominance of CD4+ T cells which are MHC Class II-restricted. In MS lesions, T cell infiltration with clonally expanded CD8+ lines has also been appreciated.[10]

Myelin Reactive T-Cells then create a cascade of inflammation through the release of cytokines. In MS lesions, it appears that a predominantly T helper type 1 response is activated mainly through interferon-γ[11]. Interleukin 12, 17, and 23 have also been shown to be mediators of the inflammatory response in MS plaques.[12,13]

Inflammatory responses activate microglial cells that are considered to be the main effectors of tissue damage in early multiple sclerosis lesions.[14] The end result of this cascade is myelin lysis, demyelination and ultimately axon transection. Axonal transection in MS plaques has been well described in active MS lesions in disease duration as short as two weeks in some cases.[15] It is likely that axonal transection is in part culprit for the incomplete resolution of MS relapses.[2]

## Biological basis of relapse symptoms

The symptoms that occur during a relapse of MS seem to be related to slowed axonal conduction and conduction block. Conduction velocity in the axolemma is dependent on the high concentration of sodium channels within the nodes of Ranvier.[16] Once demyelinated the underlying axolemma has a much reduced density of sodium channels thus not permitting saltatory conduction, and resulting in conduction slowing, conduction block and the classical symptoms of MS.[17] An alternate hypothesis suggests that auto-antibodies bind sodium channels, thus making the axolemma inexcitable and therefore producing conduction block.[18] Nitric oxide produced by glial cells within MS lesions also has been shown to directly reduce axonal conduction. Studies in humans have shown an increase in inducible Nitrogen oxide synthetase in the CSF of patients with active MS.[19] Cytokines have also been implicated in decreased axonal conduction, specifically Tumor Necrosis Factor α and Interferon-γ.[20] This also, in part, explains transient worsening of MS symptoms during fever and concurrent infection. Cytokines may also cause other non focal symptoms such as fatigue, malaise, and cognitive clouding which can also occur during a relapse.[21]

Exacerbations may also re-occur with similar clinical characteristics. Occasionally, the re-occurrence of an exacerbation occurs in a slightly more severe or anatomically more extensive region. This is due to the result of new disease activity at the rim of old lesions.

## Imaging changes in MS relapses

Gadolinium enhancement on MRI reflects the breakdown of the blood brain barrier at sites of active lesions in patients with clinical relapses.[7,22] Gadolinium enhancement occurs either homogenously or in a ring enhancing fashion. An open ring sign appears to be characteristic of MS lesions and differentiates them from tumor or abscess.[23] Enhancement typically precedes symptom onset and T2 changes.[24] Serial MRI studies have shown that lesions typically enhance for approximately 4 weeks.[25] However, similar serial studies have shown that new lesions on MRI are frequently not associated with clinical relapses, and that enhancing lesions occur more frequently than clinical relapses. (up to 5-10 times as often as clinical symptoms).[26] The exact explanation of this incongruence has not been elucidated. However it may relate to factors such as lesions in ‘noneloquent’ areas of the brain; plasticity masking lesion occurrence; primarily spinal lesions; lesions that do not affect function due to residual safety factor for transmission; or functional effects that are not noticed by the patient such as a subtle cognitive change.

## Natural course of MS relapses

As noted previously, approximately 80% of MS cases begin as a relapsing condition. During early stages of relapsing remitting MS, the frequency of relapses has been estimated at approximately one per year.[27] Recovery from a relapse is often incomplete. A large cohort study found that 17% of initial relapses were followed by incomplete recovery.[28] Individual relapses have been estimated to produce an increase of 0.24 to 0.57 points on the expanded disability status scale (EDSS) score.[29] Progression of disability seems to be increased in patients with higher number of relapses during the first and second year of the disease.[3]

Infections have been associated with the occurrence of MS relapses. It is believed that activation of immune pathways, mainly via cytokines, is responsible for the more severe occurrence of relapses during infection.[6] Stress has also been reported to increase the frequency of exacerbations. This has been confirmed in a prospective study.[30] Stress also has been linked to altered cytokines and pro-inflammatory changes which may set the stage for increased disease activity.[31]

## Treatment of MS relapses

### Rationale for treatment

The goal of treating MS relapses is to decrease the duration and intensity of neurological dysfunction. Promoting a full recovery to the baseline level of functioning and reducing resultant long term disability is to date an elusive goal. The cost of a single relapse in Multiple sclerosis has been estimated to be as high as $12 870 for medical care alone.[32] Incomplete resolution of relapses has been associated with ongoing disability.[29] Relapses also cause significant psychological stress on patients with MS.[33,34]

Treatment of relapses has been heavily guided by expert opinion and anecdotal experience.[35] We analyze the different agents for treatment of relapses with a brief description of the scientific basis of their use. Next, an evidence-based analysis of the effectiveness of these medications is presented. We use the following format for grading evidence:

- Level I: Prospective, randomized controlled clinical trial with masked outcome assessment, in a representative population.
- Level II: Prospective matched group cohort study in a representative population with masked outcome
- Level III: All other controlled trials (including well-defined natural history controls or patients serving as own controls) in a representative population, where outcome assessment is independent of patient treatment
- Level IV Evidence from uncontrolled studies, case reports, or expert opinions[36]

### Corticosteroids (CS) and ACTH

Rationale for use

The early recognition of multiple sclerosis as an auto-immune disorder led to trials of anti-inflammatory agents for MS. ACTH has been used to induce endogenous steroid release. Steroids have been used in intravenous and oral forms. Corticosteroids are potent anti-inflammatory agents that exert their actions through nongenomic and genomic mechanisms. The rapid clinical response in MS relapses is felt primarily to be a nongenomic response.[37]

Nongenomic effects are felt to result from either specific membrane receptors or through non specific proteins and membrane lipids.[38] Steroid specific receptors on cell membranes have been well described both on lymphocytes[39] as well as on neuronal cells.[40] Activation of the glucocorticoid receptor then leads to disruption of the mitochondrial membrane potential which results in apoptosis.[41] Steroid induced apoptosis of T cells, which are the main orchestrators of inflammation in the CNS, has been demonstrated in EAE.[42] Evidence also suggests that only higher doses of steroids can achieve apoptosis.[43]

It also appears that corticosteroids reduce inflammation by decreasing the migration of inflammatory cells into the CNS. Methylprednisolone has been shown to decrease expression of adhesion molecules VLA-4 and LFA-1 in patients with MS.[44] Transmigration of monocytes into the blood brain barrier has also been shown to reduce after steroid treatment.[45] Steroids also modulate the effect of matrix metalloproteinases on the disruption of the blood brain barrier.[46] CSF studies have shown a decrease in intra-thecal synthesis of IgG after steroid treatment as well.[47]

### Evidence for use of steroids

The use of steroids for treatment of MS exacerbations has been endorsed by the National MS society and the American Academy of Neurology.[48]

Level I data support the beneficial effect of corticosteroids on speed of recovery after MS relapses. Initial data from the Optic Neuritis treatment trial showed that treatment with intravenous methylprednsiolone (IVMP) 1 g daily for 3 days followed by a 21 day tapering oral steroid treatment hastened recovery compared to placebo and oral prednisone.[49] Class I evidence from a randomized controlled trial studying 51 subjects showed that IVMP provided benefit at 3 weeks and 6 weeks on disability scores as compared to placebo.[50] Two smaller Class I-II trials compared IVMP to placebo and found a short term benefit to the steroid group.[51,52] More recently meta-analytic studies have pooled data on multiple previous trials. Brusaferri *et al*,[53] analyzed data of 12 trials comparing IV steroids or ACTH to placebo and found a reduction of disability or improvement in visual acuity at 30 days (odds ratio 0.49; 95% CI 0.37-0.64). A Cochrane Database review from 2000[54] reviewed a total of 377 patients with MS from 6 different Trials. Two of the six trials studied ACTH and the remaining 4 studied Methylprednisolone. Outcomes were better at 30 days in the steroid/ACTH group (odds ratio 0.37; 95% confidence interval 0.24-0.57).

Steroids are most often used through an intravenous route; however, there is no clear evidence that IV preparations are superior. Barnes *et al*, compared an IV and oral regimen of steroids and found no significant differences between both groups.[55] A similar previous study showed that IV and oral medications at similar dose were equally effective.[56]

The optimal dose of steroids has not been clearly established. The notion that high dose steroids are needed to treat MS relapses is derived, in part, from laboratory research as detailed above.[43] A meta-analysis which gathered data from nine different trials attempted to establish clinical differences in high dose and low dose methylprednisolone (MP) treatments. MP dose was categorized as either high dose (MP >500mg/day) or low dose (MP <48 mg/day). No significant difference was found between low dose and high dose groups. An earlier report from Italy did find that High Dose Methyl Prednisolone (HDMP) was more effective than low dose methyl prednisolone (LDMP) in the short term, and found that LD MP was associated with clinical reactivation.[57] CSF IgG synthesis measurements corroborated these results. Trials examining different doses of high dose steroids have also been conducted. A comparative trial between 2 g of IVMP daily and 500 mg of IVMP daily showed better responses in the higher dose group with more sustained effects on gadolinium enhancement.[58] A single small randomized trial (Level 1) comparing night time versus daytime treatment with IV steroids showed a more rapid response with lower side effects with the night time dosing.[59]

MR imaging has also provided evidence regarding the effectiveness of steroids in relapses. Gadolinium enhancement is a marker of disruption of the blood brain barrier (BBB) and so reduction in contrast enhancement is assumed to be related to steroid induced restoration of the BBB.[60] IVMP has been shown to decrease the number of lesions with gadolinium enhancement during MS exacerbations.[61,62] The duration of steroid-related reduction in contrast enhancement is felt to last approximately 7-9 weeks.[63]

A triad of decrease in EDSS, decrease in measurements of MBP, and a decrease in number of Gadolinium enhancing lesions has been described as evidence of steroid-related reduction of inflammation during MS exacerbations.[60] The effects of steroids on CSF analysis have shown reduction in adhesion molecules, matrix metalloproteinases, neuronal degradation products, and IgG synthesis.[44,46,47,57,60]

The use of a steroid taper following IVMP is commonly prescribed and seems to be based mainly on physician or patient preference. Some data is available regarding the use of steroid tapers. A recent study compared data from 152 patients who had received oral steroid tapers and 112 who had not. No significant differences were found between the groups in follow-up.[64] There is, however, no conclusive evidence regarding the right use of steroid tapers.

### Adverse effects

Although it is clear that steroids shorten the duration of MS exacerbations, this treatment is not without adverse effects. Although inflammation seems to drive multiple sclerosis, especially during the early stages of disease, it appears inflammatory mechanisms carry out an important role in remyelination as well.[65] Animal studies have shown that steroids decrease oligodendrocyte-mediated repair of demyelinated lesions.[66]

Brain atrophy has been seen with steroid use in MS exacerbations.[67] It is however felt that the majority of brain atrophy is driven by a decrease in lesion load and lesional edema.[68] It is also clear that steroids have a detrimental effect on cognition and memory. However, it seems the effect is transient and reversible.[69,70]

Bone health has also been a potential concern with use of steroids. It seems steroids induce an immediate fall in bone formation and increase in bone resorption following a high dose of IVMP.[71] However, it appears this effect is transient. Long term studies of pulsed steroids have not shown a detrimental effect on bone mineral density.[72]

Steroids also produce significant effects in other bodily systems. Lyons *et al*,[73] meticulously examined 350 treatment courses of IVMP and found adverse effects which included hyperglycemia or glucosuria in 4.6% of treatments. GI intolerance and dyspepsia were also observed; these required therapy with H2 antagonists in some occasions. Psychiatric manifestations including euphoria and depression were also seen. Minor side effects were less common and included: taste disturbance, flushing, weight gain, paresthesias, and insomnia. The concerning side effects of repeated steroids, though rare, also include aseptic necrosis of the shoulder or hip, and the development of cataracts.

### Conclusions

Good level I and II evidence supports the use of steroids for decreasing the duration of symptoms during MS exacerbations [Table 1]. MRI and CSF parameters support these findings. IV steroids are the preferred route of administration. High doses of IV methylprednisolone, used in clinical trials show effectiveness, dosage vary from 500-1000 mg daily for 3-5 days. There is no evidence to support the use of oral steroid tapers; physician and patient preference may determine the use of these. Steroids are associated only with the transient decrease in memory and bone formation. In general, steroids should be reserved for patients having functional deficits due to an acute exacerbation to reduce the total use of steroids during their clinical course.

**Table 1:** Summary of therapies used in exacerbations

Medication	Dose recommended	Adverse effects	Level of evidence
Steroids (IVMP)	500-2000mg daily for 3-5 days	Hyperglycemia, GI Intolerance, Euphoria, Insomnia	I-II
IVIG	1-2g/kg over 4-5 days	Rash, Fever	II-III in steroid unresponsiveness
Plasma Exchange	N/A	Arrythmia, Hemolysis Myocardial infarction	II-III in steroid unresponsiveness

### Plasma exchange and IVIG

Rational for use

Given the recent recognition of B-cell immunity in the pathogenesis of MS,[74] therapies which target antibody mechanisms are logical candidates for use in exacerbations. Evidence points to antibody mediated mechanisms of symptom production during MS exacerbations as well.[18] Classically, Intravenous Immunoglobulin (IVIG) and Plasma Exchange (PE) have been considered as a second choice therapy to corticosteroids, and hence the most evident effectiveness of this therapy is in cases of steroid unresponsive relapses. The European Federation of Neurological Societies recommends use of IVIG only as a second or third line regimen for MS relapses.[75]

### Evidence for use of plasma exchange

Level III evidence for the use of PE in MS comes from a group of steroid resistant patients. Relapses improved in 71% of cases after treatment with PE. There were no controls available.[76] A crossover trial involving subjects with inflammatory demyelinating disease who had failed steroids showed clinical improvement in 42.1% of the patients receiving PE. The placebo control group of that study showed an improvement of only 5.9%.[77] The study was not composed of solely MS patients and therefore constitutes level II evidence for the use of PE in MS relapses. PE for optic neuritis unresponsive to steroids has also been studied. A retrospective review of 10 cases showed some response to PE in 7 of 10 subjects, however no controls were available and the group was not exclusively comprised of MS patients.[78] There is some level IV evidence showing the effectiveness of PE in MS relapses in secondary progressive disease as well.[79]

### Evidence for use of IVIG

An early report of IVIG suggested a high response rate (68%) to exacerbations within 24 h of treatment. It is important to note that this was a nonrandomized, nonplacebo controlled trial.[80] A trial reviewing the combined effect of IVIG and IVMP demonstrated that addition of IVIG to IVMP was no different than addition of placebo.[81] In that trial 76 patients were studied and were randomized 1:1 to either IVIG or placebo. Follow-up was conducted at four days, three weeks, 12 weeks, and 26 weeks. No significant differences were observed in both groups. The standard dose used in the above trials was 1 g/kg. A well designed randomized clinical trial of IVIG alone for treatment of MS exacerbations has yet to be conducted.

### Adverse effects

No good data exists regarding adverse effects of IVIG use in patients with MS. However, retrospective data from IVIG use in a population with predominantly neuro-muscular disease indicates that the most common non serious side effects are rash and fever.[82] In that same cohort of 1085 infusions, the rate of serious complications such as aseptic meningitis, thrombosis, hemolysis, or renal dysfunction was 0%. The rate of non serious adverse effects was 4.7%.

Plasma exchange seems to carry a higher risk of serious adverse effects such as myocardial infarction, arrhythmia and hemolysis. A retrospective review of PE in neurological disorders found that each of these adverse effects occurred only once in a study of 154 sessions of plasma exchange.[83] A German review of 291 sessions of PE in 39 patients found a 4.8% rate of minor complications and a 2.8% rate of major complications, as well as one death.[84] A large review including 385 sessions of PE found that 17% of all sessions, and almost half of the patients involved suffered some type of complication.[85] Severe complications were found in 6.5% of sessions and death occurred in 2 cases of the 63 patients. It is worth mentioning that the majority of the patients studied in these groups suffered from neuro-muscular conditions which potentially produce more hemodynamic and respiratory instability.

### Conclusions

Good level I evidence is lacking for either IVIG or PE. Level II and level III evidence supports the use of these agents in steroid unresponsive exacerbations, or patients who have a contraindication to steroids. Well-controlled randomized trials are eagerly awaited to determine the true effectiveness of both these therapies. Adverse effects for IVIG are most commonly mild and are in general less than 5%. Complications for PE occasionally can be serious (2.8-6.5%) and death is reported to occur in 1 in 30-40 patients.

## Natalizumab

### Rationale for use

The use of medications that decrease leukocyte trafficking across the blood brain barrier would seem to be ideal candidates for the treatment of MS exacerbations. Natalizumab is a humanized monoclonal antibody directed at alpha 4 beta 1 integrin. Natalizumab thus prevents binding of alpha 4 beta 1 integrin and vascular adhesion molecule 1. This reduces trafficking of lymphocytes and monocytes across the blood brain barrier.[86]

### Evidence for use

A single randomized clinical trial has evaluated the efficacy of natalizumab in the treatment of acute exacerbations in multiple sclerosis.[87] The study involved 180 patients who were randomized to either a 1 mg/kg, 3 mg/kg dose of natalizumab or placebo. Patients were followed for a total of 14 weeks following medication administration and no significant differences were found between the three groups in measurements of disability. A robust reduction in gadolinium enhancement however was found in the treatment groups. This led ultimately to further trials of pulse administration of this medication showing efficacy in prevention of new disease activity.

### Adverse effects

The above-mentioned trial found that natalizumab was well tolerated in general. The total incidence of adverse events in the placebo and natalizumab groups was similar. The most frequent adverse effects in the active medication groups were (in order of frequency): headache, pharyngitis, dizziness, and nausea.[87] The occurrence of progressive multifocal leukoencephalopathy (PML) with use of Natalizumab has been well established.[86] The initial incidence was felt to be close to 1 in 1000 cases, however as the medication has been reintroduced into the market it appears that the number may have been overestimated. It is difficult to ascertain the risk of PML if this medication were to be used for treatment of MS exacerbations; however it would likely be much less common than the above quoted rate of 1 per 1000.

### Conclusions

Evidence to date does not support the use of natalizumab for the treatment of acute exacerbations in multiple sclerosis based on results from a single randomized clinical trial. The medication was well tolerated in a single dose fashion with only minor adverse effects.

## Nonpharmacologic therapy

The importance of rehabilitation in patients with MS is well established. The use of rehabilitation during an exacerbation helps patients achieve a more complete functional status. There is evidence that planned multi-disciplinary rehabilitation along with IVMP improves short term outcomes. A blinded randomized clinical trial compared planned multi-disciplinary approach with standard ward therapy in patients receiving IVMP for an exacerbation of MS.[88] The group randomized to a planned therapy had statistically significant better outcomes at three months as compared to the control group. It appears that IVMP alone does not improve perceived health status after exacerbations, and therapy may provide some improvement in this regard.[89]

## Treatment of pseudo-exacerbations

The treatment of pseudo-exacerbations has been mainly empiric. There is little, if any, evidence to support different treatment options. When infection is suspected, it is logical to treat with appropriate antibiotic therapy. Given the relationship between fever and worsening of MS symptoms, defervescence with acetaminophen or NSAIDS is also paramount. Physical therapy during pseudo-exacerbations may also be useful in assuring prompt recovery to baseline.

## General conclusions

Exacerbations are a cardinal feature of relapsing remitting multiple sclerosis. Incomplete resolution of exacerbations is one of the main causes of early disability in the course of the disease. Exacerbations cause significant effects on both the physical and mental health of patients. This has led the medical community to treat exacerbations aggressively. However, the focus of pharmacological research has been more directed at prevention rather than treatment of exacerbations.

Only steroids have been studied in a randomized blinded, well designed fashion. It is clear from current evidence that steroids decrease duration and severity of symptoms during an exacerbation. IVMP has been the steroid most studied and has been used at doses of 500-2000 mg per day for three to five days. Steroids have not been found to be effective in decreasing long-term disability in MS. However, one must note that these studies have typically evaluated the effect of steroids on individual exacerbations. Studies assessing the long-term effect of steroids used over many years on dozens of exacerbations do not exist. Data on the use of PE and IVIG is limited to either patients who have not responded to steroids or patients who carry contraindication to steroid use. Natalizumab has not shown a beneficial effect in the treatment of exacerbations. Treatment of concurrent infections and fever is recommended on an empiric basis.
